# Immunological Memory and Affinity Maturation After Vaccination in Patients With Propionic Acidemia

**DOI:** 10.3389/fimmu.2022.774503

**Published:** 2022-03-25

**Authors:** Manuela Zlamy, Thomas Zöggeler, Magdalena Bachmann, Michael Schirmer, Christian Lechner, Miriam Michel, Alexander Schimkowitsch, Daniela Karall, Sabine Scholl-Bürgi

**Affiliations:** ^1^Department of Child and Adolescent Health, Pediatrics I, Medical University of Innsbruck, Innsbruck, Austria; ^2^University Hospital for Internal Medicine II, (Infectiology, Immunology, Pneumology and Rheumatology), Medical University of Innsbruck, Innsbruck, Austria; ^3^Department of Child and Adolescent Health, Pediatrics III, Medical University of Innsbruck, Innsbruck, Austria

**Keywords:** propionic acidemia (PA), vaccinations, relative avidity index, immune memory, antigen specific IgG concentrations

## Abstract

Earlier studies have recommended routine childhood immunization in patients with propionic acidemia (PA); however, the literature presents insufficient data on the response to vaccines, notably specific IgG concentrations and avidity maturation, after measles, mumps, rubella (MMR), and diphtheria/tetanus (DiphtTe) vaccinations in this population. In patients with PA, cellular and humoral changes of the immune system (e.g. a decreased CD4+ T cell count, with a reversal of CD4/CD8 T cell ratio, a deficient gamma-globulin fraction, and in one case a decreased lymphocyte blastogenesis) have been reported. Former reports also detected pancytopenias accompanying febrile infections in PA patients. In the current study, we analyzed vaccine-specific IgG concentrations and avidity maturation after MMR and DiphtTe vaccinations in 10 patients with PA. Compared to gender and age matched controls, all 10 had protective IgG concentrations for at least one tested antigen, and in 6 out of 10 patients high relative avidity indices for measles and rubella were detected. In summary, the present study revealed a sufficient immune response and outcome, indicating an acceptable humoral memory in patients with PA after booster vaccinations.

## Introduction

**Figure 1:**
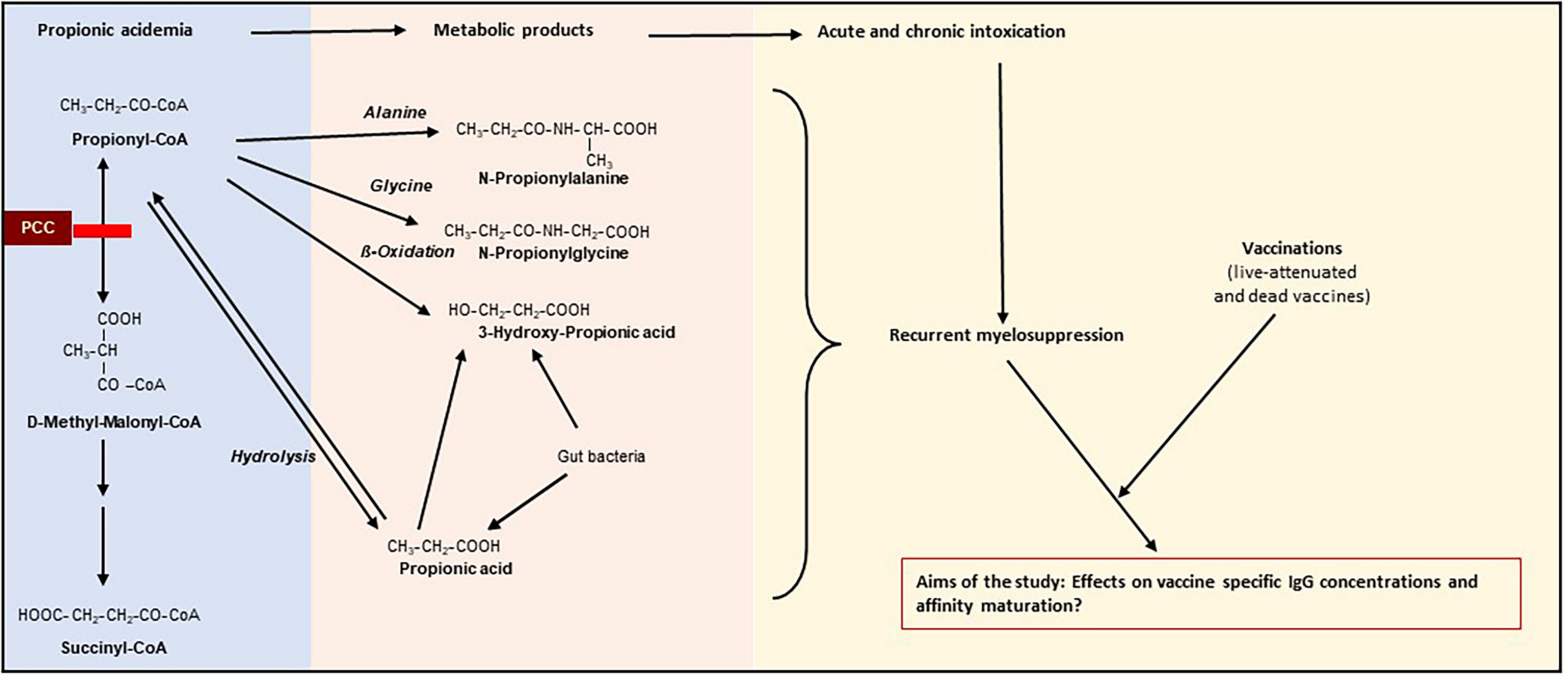
Schematic description of pathophysiology of propionic acidemia (PA) and the aims of our study [partly adapted from (1, 15)]. PCC, propionyl-CoA carboxylase; Grey line indicates blockage of PCC. Propionic acidemia is caused by an autosomal recessively inherited deficiency of the mitochondrial enzyme propionyl-CoA carboxylase, resulting in the accumulation of propionic acid and related metabolic products (e.g., propionyl CoA, propionylcarnitine, 3-hydroxy-propionic acid, propionic acid). Abnormal amounts of metabolic products result in an intoxication type disorder associated with either acute or chronic symptoms (neurological symptoms (associated with progressive encephalopathy of varying severity), gastrointestinal symptoms (failure to thrive, anorexia), hematological abnormalities (neutropenia, pancytopenia), and heart failure [prolonged QTc interval, cardiomyopathy)]. The influence of recurrent immunosuppressive episodes during early infancy on immune outcome to vaccination remains unclear.

Propionic acidemia (PA; OMIM# 606054) is a rare disease with an estimated incidence ranging from 1:166.000 (Italy) to 1:250.000 births (Germany) in western populations (1–3). PA has an autosomal recessive inheritance pattern, causing a reduced mitochondrial “propionyl-CoA carboxylase (PCC)” enzyme activity. PCC catalyzes the conversion of propionyl-CoA to methylmalonyl-CoA, which enters the Krebs cycle *via* succinyl-CoA and acts as anaplerotic substance. The enzymatic defect leads to a disrupted activity of PCC resulting in the accumulation of propionic acid and related metabolic products (e.g., propionyl CoA, methylcitrate, propionylcarnitine, propionic acid) (1, 4–14) (**Figure 1**). Abnormal amounts of metabolic products induce intoxication which lead to either acute or chronic symptoms. In the neonatal period, at around the second day of life, patients with PA present with symptoms such as acute deterioration of general condition, vomiting, dehydration, temperature instability, muscular hypo- or hypertonia, irritability, lethargy progressing to coma and seizures, and characteristic laboratory findings (persistent metabolic acidosis and ketosis, elevated anion gap and eventually hyperammonemia). The long-term sequelae mainly involve the heart and the CNS, with neurological symptoms (associated with progressive encephalopathy of varying severity), gastrointestinal symptoms (failure to thrive, anorexia), hematological (neutropenia, pancytopenia), and cardiac complications (prolonged QTc interval, arrhythmias, dilated or hypertrophic cardiomyopathy, heart failure) (1, 4–14, 16, 17). At any age, catabolic situations (e.g., during febrile episodes) can lead to acute metabolic crises associated with life-threatening events (1, 4–14).

Many countries provide newborn screening programs for the diagnosis of PA (1, 4, 18). Like other children, PA patients are exposed to different community-acquired infections of early childhood. In situations of acute metabolic decompensation, often triggered by febrile infections, PA patients are confronted with four major problems:

(1) deficiency of succinyl-CoA resulting in Krebs cycle and oxidative phosphorylation dysfunction, which leads to metabolic acidosis (1, 4, 18).

(2) the accumulation of organic acids and related metabolic products impairs the formation of N-acetylglutamate, which activates the urea cycle, and finally leads to hyperammonemia (1, 4, 18),

(3) deficient conversion of propionyl-CoA to methylmalonyl-CoA, which acts as an anaplerotic substance by being fed into the Krebs cycle (1, 4, 18) and

(4) immunosuppression with pancytopenia and increased susceptibility to sepsis (1, 4, 7, 19).

Earlier studies focusing on the immune system of patients with PA revealed cellular and humoral changes of the immune system (e.g., a decreased CD4+ T cell count, with a reversal of CD4/CD8 T - cell ratio, a deficient gamma-globulin fraction, and in one case a decreased lymphocyte blastogenesis) caused by the accumulation of organic acids and related products (8, 11, 12). It has also been shown that unusual quantities of propionic acid alter the immune function due to an impairment of immune cell maturation and proliferation (“immunosuppressive” effect of propionic acid) (8, 11, 12). The effect of metabolite accumulation on the immune system of PA patients (e.g., propionyl CoA, methylcitrate, propionylcarnitine) has not yet been investigated. However, it is currently postulated that excessive levels of propionic acid and related metabolites during catabolic crisis lead to a severe metabolic decompensation associated with recurrent states of immunosuppression, especially during the first years of life (1, 11). The overall vaccination coverage in the European population has declined and herd immunity is not always available (e.g. measles vaccination coverage <95% of inhabitants) within a population [20; Steffens I, 2015]. Therefore, active immunization in this group of pediatric patients represents the most effective strategy to decrease morbidity and mortality associated with vaccine-preventable diseases (1, 7, 11, 20, 21). Vaccine-preventable diseases (e.g. pertussis, measles) showed an increasing incidence in the Western population during the last years (e.g. measles, diphtheria) (1, 7, 11, 20, 21). However, despite the recommendations, a French study warned of lower vaccination rates and delayed administration (e.g., measles, mumps, rubella, diphtheria-tetanus-pertussis-inactivated polio) among children with inborn errors of metabolism compared to healthy controls (22).

To the best of our knowledge, no data on vaccine-specific IgG concentrations and avidity maturation after measles, mumps, rubella (MMR), and diphtheria/tetanus (DiphtTe) vaccines in patients with PA are available. The literature also lacks reports on the immunogenicity, safety, and tolerability of different kinds of vaccines (live-attenuated vaccines, inactivated vaccines) in children and adults with PA.

Therefore, we aimed to analyze the immune response and outcome of patients with PA to the live-attenuated MMR vaccine, and to the inactivated DiphtTe vaccine at different ages.

Additionally, we investigated whether patiens with PA were able to produce high avidity IgG antibodies against the inoculated antigens by selection of high-affinity virus-specific B cells.

## Results

### Baseline Characteristics

Blood samples were collected during routine visits from five male and five female PA patients. Serum was also collected to measure IgG and avidity titers. Blood samples were also collected from age- and gender- matched controls during routine appointments, such as yearly medical check-up, consultations evaluation in case of chronic cephalea, etc. Dates of vaccinations were extracted from patients’/controls’ vaccination records.

The mean age at first MMR vaccination was 4.3 ± 6.0 years (median 2.0; range 1.0-20.0) for PA patients and 2.2 ± 2.1 years (median 2.0; range 1.0-8.1) for controls. The mean age at first DiphtTe-containing vaccination was 0.6 ± 0.5 years (median 1.0; range 0.3-1.0) for the PA group and 0.4 ± 0.5 years (median 0.3; range 0.3-1.0) for the control group. Patients with PA showed similar levels of leukocyte and lymphocyte counts when compared to controls [5.3 ± 1.6 G/l (median 6.9; range 2.5-7.2) versus 5.7 ± 1.0 G/l (median 5.4; range 4.4-7.7) and 1.9 ± 0.9 G/l (median 2.1; range 0.6-3.3) versus 2.1 ± 0.3 G/l (median 2.1; range 1.6-2.7) respectively] (**Table 1**).

**Table 1:** Demographic data and characteristics of patients and controls.

		Patients with propionic acidemia (n=10)						Controls (n=10)		
Age (y)		21.5 ± 8.7 (20.4; 6.3 - 34.4)						22.0 ± 8.7 (20.7; 7.1 – 35.8)		
N.	Age (y)	Sex	Mutation*	Protein change	Gene affected	Enzyme activity	Organ involvement (CNS, Cardiac, Others)	N.	Age (years)	Sex
1	34.4	F	c.614T>A	p.Val205Asp	pccB	0.90%	CNS, Cardiac, Others	1	35.8	F
2	34.1	F	c.1118T>A	p.Met373Lys	pccA	1.20%	CNS, Cardiac, Others	2	34.6	F
3	30.5	M	c.1118T>A	p.Met373Lys	pccA	1.30%	CNS, Cardiac	3	30.4	M
4	22.5	F	c.1199-2A>G	p.(Thr401Profs*9)	pccB	0.40%	CNS, Cardiac, Others	4	23.0	F
5	21.8	M	c.614T>A	p.Val205Asp	pccB	0.80%	CNS, Cardiac	5	21.6	M
6	19.1	M	c.614T>A	p.Val205Asp	pccB	n.d.	CNS, Cardiac, Others	6	19.8	M
7	17.0	M	c.614T>A	p.Val205Asp	pccB	n.d.	CNS, Cardiac, Others	7	17.6	M
8	16.3	F	c.1333A>G	p.Lys445Glu	pccB	n.d.	Others	8	14.9	F
9	12.9	M	c.1118T>A	p.Met373Lys	pccA	n.d.	Cardiac, Others	9	15.1	M
10	6.3	F	c.966+1G>A	p.(Ser295Argfs*26)	pccB	0.10%	Others	10	7.1	F
Age first MMR vac. (years)		4.3 ± 6.4 (2.0; 1.0 - 20.0)						2.2 ± 2.2 (2.0; 1.0 – 8.0)		
Age first DiphTe vac. (years)		0.6 ± 0.5 (1.0; 0.3 - 1.0)						0.4 ± 0.5 (0.3; 0.3 – 1.0)		
Leucocytes (g/L)		5.3 ± 1.7 (5.2; 2.5 - 7.2)						5.7 ± 1.0 (5.4; 4.4 - 7.7)		
Lymphocytes (g/L)		1.9 ± 0.9 (1.8; 0.6 - 3.3)						2.1 ± 0.4 (2.1; 1.6 - 2.7)		

Average values are presented as: mean ± one standard deviation (median; range).; y, years; N, number; F, female; M, male; n.a., not applicable; pccB, propionyl-CoA carboxylase B gene; pccA, propionyl-CoA carboxylase A gene; Diph, diphteria; Te, tetanus; MMR, measles, mumps, rubella; vac, vaccination; n.d., not done; Organ involvement is indicated by: CNS, central nervous system (e.g. epilepsy); Cardiac involvement (e.g. long QTc, cardiomyopathy); other organ systems (e.g. microcytic normochromic anemia, cachexia); *All patients are homozygous for the mutation.

### Specific IgG Antibody Concentrations

**Figure 2:**
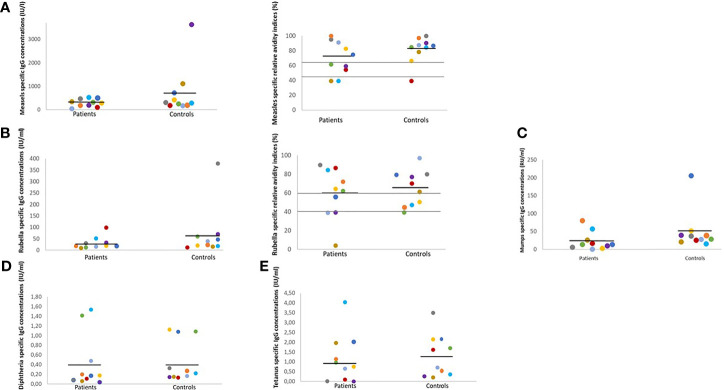
Antigen-specific IgG concentrations and RAI in sex- and age-matched patients and controls. RAI, relative avidity indices, IU, international units; RU, relative units; U, units; ml, milliliter; Each color indicates one matched pair of patient and control: pair 1 grey, pair 2 lilac, pair 3 yellow, pair 4 green, pair 5 orange, pair 6 red, pair 7 light blue, pair 8 dark blue, pair 9 brown, pair 10 violet. Black line indicates mean; grey lines indicate upper and lower RAI reference values. In age- and sex-matched patient/control pairs lower IgG concentrations and RAI rates were detected in PA patients. **(A)** Positive measles-specific IgG concentrations values ≥275 IU/l, negative IgG concentrations ≤200 IU/l. High RAI ≥60.0%, intermediate RAI 40-59.9%, low RAI ≤39.9%. **(B)** Positive rubella-specific IgG concentrations values ≥11 IU/ml, negative rubella-specific IgG concentrations ≤8 IU/ml. High RAI ≥60.0%, intermediate RAI 40-59.9%, low RAI ≤39.9%. **(C)** Positive mumps-specific IgG concentrations values ≥22 RU/ml, negative IgG concentrations ≤16 RU/l. **(D)** Positive diphtheria-specific IgG concentrations values ≥0.1 IU/ml, negative diphtheria-specific IgG concentrations ≤0.01 IU/ml. **(E)** Positive tetanus-specific IgG concentrations values ≥0.5 IU/ml, negative tetanus-specific IgG concentrations ≤0.1.

Patients with PA showed a protective antigen concentration in 64% of tested antigen-specific IgG, and at least one out of six protective vaccine-specific IgG antibody concentrations was detected several years after the last vaccination. Patients with PA had lower, however statistically not significant, antibody concentrations for all measured antigen-specific IgG concentrations compared to controls (**Figure 2**, **Table 2**).

**Table 2:** Detailed comparison of IgG specific antibody concentrations, RAI and time since last vaccination in paired age- and sex-matched patients and controls.

N.	Colour	Measles			Rubella			Mumps		Diphtheria		Tetanus	
		IgG	RAI	V.	IgG	RAI	V.	IgG	V.	IgG	V.	IgG	V.
P1	grey	**472.52^#^**	95.2^#^	n.a.	31.20^#^	**89.6**^#^	n.a.	5.74*	n.a.	0.08^+^	n.a.	0.02*	n.a.
C1		306.59^#^	**100.0^#^**	9	**378.38^#^**	79.7^#^	9	**36.80^#^**	9	**0.33^#^**	1	**3.49^#^**	1
P2	lillac	44.82*	**91.3^#^**	14	15.71^#^	38.9	14	0.93*	14	**0.48^#^**	27	**0.65^#^**	27
C2		172.83*	87.7^#^	22	**40.10^#^**	**97.0^#^**	22	**27.85**^#^	22	0.16^#^	10	0.54^#^	10
P3	yellow	281.71^#^	**82.7^#^**	n.a.	19.34^#^	**64.1^#^**	n.a.	2.89*	n.a.	0.18^#^	4	0.75^#^	4
C3		**416.97^#^**	66.5^#^	30	**20.49^#^**	50.2^+^	30	**51.68^#^**	30	**1.13^#^**	15	**2.15^#^**	15
P4	green	**322.94^#^**	61.5^#^	15	12.31^#^	**61.9^#^**	15	13.95*	15	**1.41^#^**	7	0.96^#^	7
C4		254.69^+^	**84.8^#^**	16	**61.15^#^**	39.0^*^	16	**28.14^#^**	16	1.09^#^	9	**1.70^#^**	9
P5	orange	176.12*	**100.0^#^**	17	18.62^#^	**71.7^#^**	17	**80.36^#^**	17	0.20^#^	14	**1.14^#^**	14
C5		**201.27^+^**	97.2^#^	14	**23.54^#^**	44.6^+^	14	38.93^#^	14	**0.28^#^**	5	0.71^#^	5
P6	red	105.50*	**54.4^+^**	14	**99.27^#^**	**86.3^#^**	14	17.18^+^	14	0.11^#^	18	0.10^#^	18
C6		183.90*	39.0*	13	12.57^#^	70.0^#^	13	**25.28^#^**	13	**0.13^#^**	5	**1.61^#^**	5
P7	light blue	**528.74^#^**	39.0*	15	**52.23^#^**	**84.1^#^**	15	**56.90^#^**	15	**1.54^#^**	2	**4.04^#^**	2
C7		287.27^#^	**84.3^#^**	10	18.11^#^	47.0^+^	10	15.58*	10	0.22^#^	2	0.36**^#^**	2
P8	dark blue	505.85^#^	74.6^#^	12	19.51^#^	55.9^+^	12	13.92*	12	0.17^#^	1	2.02^#^	1
C8		**724.25^#^**	**86.9^#^**	12	**46.85^#^**	**79.2^#^**	12	**205.69^#^**	12	**1.08^#^**	7	**2.16^#^**	7
P9	brown	344.54^#^	39.0*	7	9.44^+^	3.8*	7	**26.37^#^**	7	0.06^+^	5	**1.97^#^**	5
C9		**1105.89^#^**	**78.2^#^**	13	**15.81^#^**	**61.0^#^**	13	20.97^+^	13	**0.15^#^**	7	0.21**^#^**	7
P10	violet	192.40*	59.2^+^	5	33.95^#^	39.0*	5	9.68*	5	0.04^+^	6	0.00*	6
C10		**3627.83^#^**	**90.2^#^**	n.a.	**71.13^#^**	**76.8^#^**	n.a.	**39.69^#^**	n.a.	**0.14^#^**	6	**0.26^#^**	6
Patients													
pos		**60**	**60**		90	**60**		30		70		70	
neg		40	20		0	30		60		0		20	
borderl		0	20		10	10		10		30		10	
Controls													
pos		60	**90**		**100**	**60**		**80**		**100**		**100**	
neg		20	10		0	10		10		0		0	
borderl		20	0		0	30		10		0		0	

PA patients showed a protective antigen concentration in 64% of tested antigen specific IgG concentration. 60% of PA patients showed high measles and/or rubella specific RAI. In age- and sex-matched patient/control pairs an individual immune outcome were detected to all evaluated tested antigens and RAI. A trend to a diminished immune outcome was only detected in the youngest PA patient (pair 10).

N., number; IgG, IgG concentrations; RAI, relative avidity indices; V., time since last vaccination (years); IU, international units; RU, relavite units; l, litre; ml, millilitre; n.a., not available; pos, positive IgG concentrations (% of all patients or controls); neg, negative IgG concentrations (% of all patients or controls); border, borderline IgG concentrations (% of all patients or controls);

Positive^#^ measles specific IgG concentrations values ≥275 IU/l, negative* IgG concentrations ≤200 IU/l. High RAI ≥60.0%, intermediate RAI 40-59.9%, low RAI ≤39.9%. Positive^#^ rubella specific IgG concentrations values ≥11 IU/ml, negative* rubella specific IgG concentrations ≤8 IU/ml. High RAI ≥60.0%, intermediate RAI 40-59.9%, low RAI ≤39.9%. Positive^#^ mumps specific IgG concentrations values ≥22 RU/ml, negative* IgG concentrations ≤16 RU/l. Positive^#^ diphtheria specific IgG concentrations values ≥0.1 IU/ml, negative* diphtheria specific IgG concentrations ≤0.01 IU/ml. Positive^#^ tetanus specific IgG concentrations values ≥0.5 IU/ml, negative* tetanus specific IgG concentrations ≤0.1 ^+^ indicates borderline concentrations; Positive concentrations are indicated by green background, borderline concentrations are indicated by yellow background, negative concentrations are indicated by red background. The highest concentration of each pair is indicated by bold face.

Six out of 10 participants in both groups had protective measles-specific IgG antibody concentrations. Nine out of ten patients and all the controls showed positive rubella-specific IgG antibody concentrations. Protective mumps-specific IgG antibody concentrations were detected in three out of ten patients and eight out of ten controls. Seven out of ten patients and all the control subjects showed protective diphtheria-specific IgG antibody concentrations. Lastly, seven out of ten patients and all the controls showed protective tetanus-specific IgG antibody concentrations (**Figure 2**, **Table 2**).

In summary, we did not find any significant differences in IgG concentrations between the two groups. Furthermore, no correlations for age, age at last vaccination, time since last vaccination, and specific IgG concentrations were found.

### Relative Avidity Indices (RAI)

Six out of ten patients with PA showed high (>60%) measles- or rubella-specific RAI. However, protective antigen-specific IgG concentrations were not associated with high RAI in patients with PA or controls (**Figure 1**, **Table 2**).

No consistent findings in regards to RAI could be found in the patient/control pairs: some patients with PA had either lower or higher measles- and rubella-specific RAIs compared to their controls, whilst in some pairs no significant differences between patient/control could be found (**Figure 2**, **Table 2**).

No significant correlations could be found for age, age at last vaccination, time since last vaccination and specific RAI in patients and controls.

### Individual Response to Vaccination in Age- and Sex-Matched Patient/Control Pairs

Each age- and sex-matched patient/control pair developed immune outcome for each of the five tested antigens. We observed higher antigen-specific IgG concentrations or RAI in some patients when compared to controls. A weak immune outcome was only detected in our youngest PA patient (pair 10) for all five tested antigens (**Figure 2**, **Table 2**).

Pair 1 (**Figure 2**, grey dots): The rubella-specific RAI was higher in patient 1 compared to control.

Pair 2 (**Figure 2**, lilac dots): Neither patient 2 nor control 2 had positive measles-specific IgG concentrations or rubella specific RAI.

Pair 3 (**Figure 2**, yellow dots): Patient 3 had a higher rubella-specific RAI in contrast to control.

Pair 4 (**Figure 2**, green dots): Positive measles-specific IgG concentrations and high rubella-specific RAI were detected in patient 4 in contrast to control 4.

Pair 5 (**Figure 2**, orange dots): In patient 5, tests revealed high measles- and rubella-specific RAI. Control 5 had only high measles-specific RAI.

Pair 6 (**Figure 2**, red dots): Both patient and control 6 showed high rubella-specific RAI.

Pair 7 (**Figure 2**, light blue dots): In patient 7 results revealed positive IgG concentrations for all tested antigens, and a high rubella-specific RAI.

Pair 8 (**Figure 2**, dark blue dots): In contrast to patient 8, control 8 showed positive IgG concentrations for all tested antigens and RAI.

Pair 9 (**Figure 2**, brown dots): We found positive measles-, mumps- and tetanus-specific IgG concentrations in patient 9.

Pair 10 (**Figure 2**, violet dots): Patient 10 had positive rubella-specific IgG concentrations. In control 10, positive IgG concentrations were detected for all tested antigens and RAI. Patient 10 was hospitalized four times due to metabolic decompensations following infections, and once due to a scheduled operation (metabolic stress) during the first five years of life. The metabolic decompensation episodes were not correlated to the vaccinations.

## Discussion

To the best of our knowledge, this is the first prospective study focusing on vaccine-specific IgG antibody concentrations and corresponding relative avidity index (RAI) in patients with PA and matched healthy controls. The effectiveness of vaccinations is routinely monitored by assessing vaccine-antigen specific IgG antibody levels and the corresponding RAI in formerly immunized probands (23–29). For patients with PA, the current recommendation is to be vaccinated against MMR and DiphtTe vaccines according to the national vaccination schedules, with booster doses every 10 years (30). Accordingly, all patients and controls in our study cohort received MMR and DiphtTe vaccinations during childhood, however, some patients and controls did not receive booster doses.

### Baseline Characteristics

At the time of the first vaccination, PA patients’ mean age was slightly higher than controls. A French study found suboptimal vaccination coverage among infants with inherited metabolic disorders (IMD) and exceptionally low live-attenuated vaccines coverage rates in IMD patients with concomitant immunodeficiencies (22). Other studies have shown similar vaccination rates between children with IMD and healthy peers based on the time of vaccine administration during childhood (8, 31). Generally, patients with IMD receive all vaccinations as recommended; however, the first vaccination is typically delayed. Our data corroborate these findings and suggest that the reason behind the delay might be the parents’ decision to reschedule due to recurrent febrile infections.

### Specific IgG Antibody Concentrations

Regarding the production of antigen-specific IgG antibodies after contact with neo-antigens (e.g., vaccinations), all tested patients with PA showed immune protection for at least one antigen (either non-replicating protein antigens or live-attenuated antigens). Interestingly, patient 2 had positive rubella-specific IgG values 14 years after the last vaccination and positive diphtheria- and tetanus-specific IgG concentrations 27 years after the last vaccination. A life-long protective IgG concentration has been reported after vaccination with live-attenuated MMR vaccine and DiphtTe-containing vaccine in immune-healthy individuals (mathematically calculated protection > 200 years against measles and mumps, 19 years half-life estimates against diphtheria, and 11 years against tetanus) (32). Our results are in line with the observation that immune-healthy individuals maintain long-lived B-cell memory (32).

Different types of immune responses take place after vaccination depending on the type of vaccine used: live-attenuated vaccines (e.g., MMR) induce a predominant T cell-dependent immune response, whereas inactivated vaccines (e.g., DiphtTe-containing vaccines) stimulate a different immunological memory (23–27, 33). In line with these findings, some patients with PA and controls had protective diphtheria/tetanus-specific IgG concentrations and negative MMR-specific IgG concentrations.

Similar to our findings, a study focusing on antibody maturation after trivalent inactivated influenza vaccine in children with IMD (amino acid disorders, glycogen storage disease type I, methylmalonic aciduria) showed that the immune response of patients with IMD is comparable to that of healthy age- and sex-matched controls (33).

Reports highlighted a statistically significant correlation between high tetanus antibody concentrations and age progression in healthy children (34, 35). Eight of our patients with PA (80%) showed protective tetanus-specific IgG concentrations. Interestingly, our youngest patient (patient 10) had negative tetanus-specific IgG concentrations six years after the last vaccinations, whilst control 10 had positive IgG concentrations. This discrepancy might be explained by recurrent febrile infections and the concomitant, subsequent immunosuppression during the vaccination period in the first years of life. It is not known if the immune system of PA patients is stable enough to maintain an immune response to the first vaccinations of a primary immunization schedule, and if the vaccination is associated with febrile infections causing exacerbations and linked episodes of immunosuppression. Our study is a single observation that requires further in-depth analysis to focus on the influence of recurrent immunosuppression on the primary immune response to vaccinations.

We did not find any influence of age, sex, and number of consecutive vaccine doses on the five tested IgG concentrations. Although our results are limited to our study population, older patients with PA who received booster vaccinations later in life showed a sufficient humoral immune outcome, indicating an acceptable humoral memory.

### Relative Avidity Indices (RAI)

Focusing on long-term humoral immune response after vaccination, RAI has proven to be a reliable marker (23, 28). Interestingly, 60% of our PA patients showed higher measles- and rubella-specific RAI compared to age- and sex-matched controls. The lack of RAI in patients 7 and 9 indicate an altered immune priming despite the protective antigen-specific IgG concentrations. One possible explanation may be the weak affinity maturation due to a reduced number of CD4 T cells and the consequent inadequate help for B cell maturation (23, 28).

### Limitations of Our Study

The small sample size is a major limitation of our study.

Consequently, we could not determine the influence of age, sex, and leukocyte counts, or include a multiple regression analysis to describe the relationship between the response variable (vaccine-specific IgG concentrations and RAI) and the candidate explanatory variables (e.g., age at blood withdrawal, sex). Due to the heterogenous age distribution of our study population, in most cases no data on exacerbations due to febrile infections in the first years of life are available. Therefore, we could not find a link between an acute exacerbation (including immunosupression) during early childhood and the low RAI.

## Conclusion

Our study revealed that patients with PA show protective vaccine-specific IgG concentrations and RAI after vaccination with live-attenuated and inactivated vaccines. Adolescent and adult patients with PA showed a sufficient humoral immune outcome several years after the last vaccinations, indicating an acceptable induction of a humoral memory. We therefore recommend to vaccinate patients following the national vaccination schedules, including booster vaccinations.

Evaluating PA patients’ immune outcome remains the best strategy to detect a delayed primary response, which might curb memory maturation later in life. We recommend state-of-the-art tools of assessing antigen-specific T and B cell responses utilizing TCR and BCR repertoire and immunoglobulin-Seq with LC/MS to identify predominant clonotypes along with conventional flowcytometric analyses and ELISPOT assays.

Additionally, more extensive longitudinal studies on age-stratified PA patient groups are necessary to evaluate whether it is necessary to update current vaccination guidelines by age groups or not.

## Materials and Methods

### Study Population

We prospectively enrolled 10 patients with PA (mean age 21.5 ± 8.7 (median 20.4; range 6.3 - 34.4) years) and 10 healthy age- and sex-matched controls (mean age 22.0 ± 8.7 (median 20.7; range 7.1 – 35.8) years) to evaluate 1) measles-, mumps-, rubella-, diphtheria-, and tetanus-specific (MMR) IgG antibody concentrations and 2) measles- and rubella-specific IgG antibody avidity indices (**Table 1**). All patients and controls had received MMR and diphtheria/tetanus vaccines prior to this study.

Data concerning the time of MMR vaccination administration and doses were available for eight patients and nine controls, while records on time of administration were missing for two patients and one control.

Seven patients and ten controls had received two MMR vaccine doses. Control 1 was offered revaccination with a single MMR vaccine dose nine years earlier, aged 26.

Data on the time of diphtheria/tetanus vaccinations administration and doses were available for nine patients and ten controls.

Nine patients and 10 controls had received three doses of diphtheria/tetanus vaccine in their first year of life, and five patients and nine controls received booster vaccinations as per recommendation (**Table 1**).

Neither PA patients nor controls suffered from allergic, autoimmune, or known hematologic diseases, underwent transplantation, or received immunosuppressants or cortisone. None of the subjects had infections in the two weeks preceding blood sampling or received any pharmacologic treatment known to influence blood production in the bone marrow or the immune system in the six months prior to blood sampling. No PA patient suffered from acute metabolic decompensation. Due to the small sample size, we could not observe gender-related differences in antigen-specific IgG concentrations and related RAI.

This study was conducted according to Good Clinical Practice guidelines and the Declaration of Helsinki 2011 and was approved by the local Ethics Committee (nr. 1152/2018, Medical University Innsbruck, Austria). All patients or parents and controls gave written informed consent. Both MMR and diphtheria/tetanus vaccines are routinely recommended in the Austrian Health and Vaccination Schedule.

### Antibody Assay

Detection of IgG antibody concentrations against MMR, diphtheria, and tetanus was performed using the commercially available ELISA (Euroimmun, Luebeck, Germany) according to the manufacturer’s manual. Antibody concentrations were calculated in International Units (IU)/ml for measles, rubella, diphtheria, and tetanus and Relative Units (RU)/ml for mumps. Accordingly, measles-specific IgG levels were calculated in IU/liter. All antibody concentrations were interpreted according to the instructions in the manufacturer’s manual. Antibody concentrations higher than the upper reference limit indicated by the manufacturer were diluted 400-fold and recalculated. As protective antibody levels were defined by using specific reference values recommended by the manufacturer. The cut-off values for protective antibody levels have been defined in earlier studies. The protective concentrations therefore correlate with prevention of disease and differs quantitatively and qualitatively, depending on the vaccine used (36).

ELISA-specific reference values:

•Measles:

 ∘positive - specific IgG concentrations values ≥275 IU/l

 ∘negative - specific IgG concentrations ≤200 IU/l

 ∘borderline - ≥199 IU/l - ≤ 274 IU/l

•Mumps:

 ∘positive - specific IgG concentrations values ≥22 RU/ml

 ∘negative - specific IgG concentrations ≤16 RU/ml

 ∘borderline - ≥17 IU/l - ≤ 21 RU/ml

•Rubella:

 ∘positive - specific IgG concentrations values ≥11 IU/ml

 ∘negative - specific IgG concentrations ≤8 IU/ml

 ∘Borderline - ≥9 IU/l - ≤ 10 IU/ml

•Diphtheria:

 ∘positive - specific IgG concentrations values ≥0.1 IU/ml

 ∘negative - specific IgG concentrations ≤0.01 IU/ml

 ∘borderline - ≥0.02 IU/l - ≤ 0.09 IU/ml

•Tetanus:

 ∘positive - specific IgG concentrations values ≥0.5 IU/ml

 ∘negative - specific IgG concentrations ≤0.1 IU/ml

 ∘borderline - ≥0.2 IU/l - ≤ 0.4 IU/ml

### Avidity Assay

*Antibody avidity* refers to the total non-covalent interaction between a specific antibody and its antigen. A high antibody avidity results from the somatic hypermutation and affinity-based selection of antigen-specific B cells in the germinal centers of secondary lymphoid tissues (21). A high relative antibody avidity index is generated by the high stability of the antigen-antibody-complex in the presence of urea, which induces dissociation. The measles- and rubella-specific IgG antibody avidities were tested with commercially available ELISA kits using urea as a dissociating agent. The relative avidity index (RAI) was calculated after measuring the extinction of a sample treated with urea multiplied by factor 100 and divided by the extinction of the baseline sample, not pretreated with urea. Data were interpreted according to the manufacturer’s instructions. Low measles- or rubella-specific IgG antibody avidity was defined by RAI values <40%, high measles- or rubella-specific IgG antibody avidity was defined by RAI values >60%, and values between 40-60% were defined as intermediate RAI (23).

### Statistical Analysis

GraphPadPrism for Windows version 9.0.0 (^®^GraphPad Software LLC, San Diego, CA, USA) was used for statistical analysis. The Mann-Whitney-U test was used to compare PA patients with controls. A p-value ≤ 0.05 was considered as statistically significant. Average values are presented as mean ± one standard deviation (median; range).

## Data Availability Statement

The blinded raw data supporting the conclusion of this article will be made available by the authors upon request, following the General Data Protection Regulation.

## Ethics Statement

The studies involving human participants were reviewed and approved by Ethics Committee (Medical University Innsbruck, Austria). Written informed consent to participate in this study was provided by the participants’ legal guardian/next of kin.

## Author Contributions

MZ designed the study, examined patients, prepared samples, analyzed and interpreted results, and wrote the manuscript. TZ, MB, and AS examined patients, prepared samples, and analyzed results. CL examined patients and prepared samples. SS-B, DK, MM, and MS examined patients, and assisted in analysis of results. All authors proofread the manuscript and approved the final version of the manuscript.

## Conflict of Interest

This work is part of the project “Inherited Metabolic Disorders and Immune Functions” supported by a financial grant from the ÖGKJ (Austrian Society of Pediatrics and Adolescent Medicine).

The authors declare that the research was conducted in the absence of any commercial or financial relationships that could be construed as a potential conflict of interest.

## Publisher’s Note

All claims expressed in this article are solely those of the authors and do not necessarily represent those of their affiliated organizations, or those of the publisher, the editors and the reviewers. Any product that may be evaluated in this article, or claim that may be made by its manufacturer, is not guaranteed or endorsed by the publisher.
